# Meeting Malaria

**DOI:** 10.4269/ajtmh.19-0778

**Published:** 2020-03

**Authors:** Emma de Sousa

**Affiliations:** Trinity College Dublin, School of Medicine, Dublin, Republic of Ireland

Arriving in the pediatrics department, I realized my expectations of medicine in Uganda were worlds away from reality. I had agreed to go on call with the pediatric intern that evening. “Just for emergencies” he had said, which had given me the image of sitting in the ward waiting for any acutely unwell children who may or may not appear. On reaching the ward, I was instead met by more than 50 mothers with children waiting at the emergency bench to see the one doctor.

Our first patient had blood results with them, showing a blood smear positive for malaria and a hemoglobin of 3.1 g/dL, the lowest hemoglobin I had ever seen and one which requires swift transfusion. A femoral tap was carried out immediately for blood grouping and crossmatch, without hesitation or any attempts at cannulation for fear of wasting time. The intern took me to the laboratory, showed me how to sign in our samples, who to give them to, and then left me there, so he could return to the queue on the ward. I waited for the blood to be analyzed, feeling considerably out of my depth but trying to appear confident. “He’s O+” said the laboratory technician “and I’m sorry, we don’t have any.” I was stunned that one of the biggest hospitals in Uganda could just run out of blood. I thought this was going to be some sort of crisis, and people on the ward would be appalled that running out of O+ could possibly be allowed to happen. “Do you have any O−?” I asked. “We have just one bag, but if you really need it then you can take it.” As medical students in Europe, we have absolutely no responsibility for decision-making and are heavily supervised even through the early years of our careers; and now, someone was asking me to decide whether to take the last unit of the universal donor from a bank supplying a whole hospital. Deciding I was definitely not qualified to make this decision, I ran to the ward and asked the intern what to do. Looking at me like I had just fallen from the sky, he said “of course, the hemoglobin is 3.1, he will die without it.” And that was that, I emptied the blood bank on my first day in the hospital.

I quickly learned that the situation on that first night was not uncommon; it was the norm. Every night, at least five children younger than 5 years came in with malaria and a hemoglobin below 5 g/dL, and most nights, the blood bank ran dry. This meant that relatives had to donate to keep their children alive, but the blood groups are not always compatible or the relative is HIV+. Almost every night, some children do not receive the transfusions they need and some of them die for this reason.

“How much malaria had you seen before coming here?” one of the interns asked during my second week. I told him I’d never seen it before and that we don’t really have it where I’m from.

“There’s whole hospitals without any malaria?” he questioned me further, sounding skeptical.

“Yes, there’s whole countries, virtually a whole continent,” I replied.

Unconvinced, he continued, “So what’s your malaria? What kills the most children where you’re from?” This was another wake-up call to me; a world without malaria, a world without children dying every night, was unfathomable to him.

I kept pushing myself on through the work on the ward, trying to keep up with the interns. They work 7 days a week plus two nights, resulting in levels of tiredness that are incompatible with providing the best care for patients or themselves. By my third week, I had seen more, knew more, and was less shocked by the things on the ward; then I had an evening call shift, which will forever remain in my mind.

Arriving on the ward at 8 pm, I was met by the medical officer. He said, “Put on gloves, this one bites.” We restrained the struggling child to take blood, and I was sent to the laboratory to get a group and crossmatch, and bring back the transfusion. She was so clinically anemic; we did not need the hemoglobin result to know that transfusion was essential to her survival. It took 20 minutes for the blood to be confirmed as compatible; when I returned to the ward, the 3-year-old girl was already dead. The full blood count and blood film confirmed malaria and a hemoglobin of 2.6 g/dL. I returned the unused blood to the laboratory, utterly dejected, but there was no time to stop to process this. Work does not, and cannot, pause here when you lose a patient.

Ten minutes later, the intern asked me to check a patient’s vitals. The boy was 4 years old, with a diagnosis of cerebral malaria. He was not breathing, sats were not registering; he did not respond to a sternal rub, and his pupils were dilated and not reactive to light. I knew what this meant, but I could not tell the mother who is anxiously looking up at me with her eyes full of fear. I told the intern. He came and spoke a few soft words in the local language. The mother began to cry, and a mix of rage and sadness rose in me at the injustice of it. How is it possible that in the same world where babies born at 24-weeks gestation can survive and technology such as extracorporeal membrane oxygenation exists that a child still dies every 2 minutes from this preventable disease?

My final malaria lesson was a personal one, hailed by a combination of fever, freezing chills, the worst headache I have ever experienced, a variety of miserable GI symptoms, and the very real concern I may not physically be able to fly home. On my last night in Uganda, I got a first-hand reminder that atovaquone/proguanil is only 90% effective as prophylaxis. Being infected with this monstrosity of a parasite is not an experience I would ever like to relive, but I am thankful that I am a healthy nonpregnant 23-year-old and I came out the other side of it with no major complications, a happy ending for me.

The story of malaria in Uganda, however, is far from its happy ending. In 2017, according to the WHO, the country had an estimated 8.6 million cases and 14,400 deaths, accounting for 4% of the global malaria burden. The impact of malaria is clear when you look at these statistics, but what I failed to understand before experiencing it is the real human cost. I saw the humanity of it in the frightened eyes of children struggling to breathe, the desperation of parents helplessly watching their child seize, the misery of those who lost their children, and the exhausted faces of the doctors who cannot do any more than attempt to put out each fire as it comes through the door.

Humanity’s fight against malaria has spanned decades, and objectively, progress has been made. However, the first time I met malaria, it felt like the disease was winning. We must not forget how much work is still needed and what is at stake.

We cannot relent.

